# Influence of lateral single jets for thermal protection of reentry nose cone with multi-row disk spike at hypersonic flow: computational study

**DOI:** 10.1038/s41598-023-33739-2

**Published:** 2023-04-21

**Authors:** Yunbin Shi, Qiong Cheng, As’ad Alizadeh, Hongbo Yan, Gautam Choubey, K. Fallah, Mahmoud Shamsborhan

**Affiliations:** 1 Geely Automotive Institute, Hangzhou Vocational & Technical College, Hangzhou, 310018 China; 2 Department of Civil Engineering, College of Engineering, Cihan University-Erbil, Erbīl, Iraq; 3 Geely Automobile Research Institute (Ningbo) Co., Ltd., Ningbo, 315336 China; 4 Department of Mechanical & Aerospace Engineering, Institute of Infrastructure Technology Research and Management (IITRAM), Ahmedabad, Gujarat 380026 India; 5 Department of Mechanical Engineering, Semnan University, Semnan, Iran; 6 Department of Mechanical Engineering, College of Engineering, University of Zakho, Zakho, Iraq

**Keywords:** Aerospace engineering, Mechanical engineering

## Abstract

The main challenge for the advancement of current high-speed automotives is aerodynamic heating. In this study, the application of lateral jet for thermal protection of the high-speed automotives is extensively studied. The simulation of the lateral coolant jet is done via Computational fluid dynamic at high-velocity condition. Finding optimum jet configuration for reduction of the aerodynamic heating is the main goal of this research. Two different coolant jets (Helium and Carbon dioxide) are investigated as coolant jet and flow study and fuel penetration mechanism are fully presented. In addition, the thermal load on the main body of nose cone is compared for different configurations. Our results specify that the injection of lateral jet near the tip of spike is effective for thermal protection of main body via deflection of bow shock. Also, Carbon dioxide jet with lower diffusivity is more effective for the protection of forebody with multi-row disk from sever aerodynamic heating.

## Introduction

In the aerospace and automotive context, Aerodynamic heating is known as the process of heating near the solid body due to change of the hypersonic/supersonic flow into energy term^1,2^. Although it seems that the transformation of momentum into thermal energy is simple process, its impacts on the flow are highly complicated^3–5^. The process of aerodynamic heating mainly happens near the nose cone of high-speed automotives. This process is highly significant for these high-speed automotives and it influences on the burning of the nose cone because of the splendid heating^6–9^. In addition, aerodynamic heating results in the noise for transmission of digital signal. These disadvantageous of aerodynamic heating have motivated the aerospace and automotive engineers to manage this process^10–12^.

There are several techniques for protection of the nose cone from aerodynamic heating. The main challenge for managing of aerodynamic heating is drag force^13–15^. In fact, drag force level should be kept in the recommended techniques. Three main techniques of mechanical, fluidic and energy devices have been investigated and examined in the previous works^16–19^. In these techniques, spike, coolant jet and energy source are used, respectively, to avoid attachment of the free stream to the main body. These techniques could efficiently reduce the temperature of the main stream after receiving to the main body^20–22^. However, the main challenge for these techniques is high drag force and this is the topic of the researchers to resolve this problem in this field^23,24^.

Among these methods, the main conventional technique for reduction of high heat load near the nose cone is spike^25–28^. Spike is known as the long thin rod located at tip of nose cone to deflect the main supersonic flow from main nose cone^29,30^. The usage of spike as a practical method is due to its simplicity^31,32^. Besides, the drag force is reduced in this technique since the supersonic air stream is bifurcated by spike. The shape of spike tip and length of spike is known as two effective factors on the performance of this technique. Previous researches^33–37^ showed that the cooling performance of this technique is not acceptable as drag force although limited thermal load reduction is reported by the application of the spike. Therefore, investigations have focused on new techniques which could compensate this deficiency of mechanical technique^38–40^. Theoretical approaches^41–47^, i.e. computational fluid dynamic, as well as experimental technique enables the researchers to improve their investigations in inaccessible conditions^48–55^. Thus these techniques are extensively used in engineering applications^55–61^.

Hybrid techniques have been recently investigated as new approach for the drag and heat reduction on the nose cone flying at hypersonic speed^62,63^. In this methodology, spike is joint with either fluidic and energy methods to improve the performance of classical technique of mechanical methods^64–67^. Although this approach seems very efficient, it is not considered as practical method yet. In fact, the usage of either fluidic and energy device for thermal load reduction is done in the laboratory and no real practical applications of this method was not reported. Since this hybrid method was new method, limited resources and articles have been presented in this topic.

**Figure 1:**
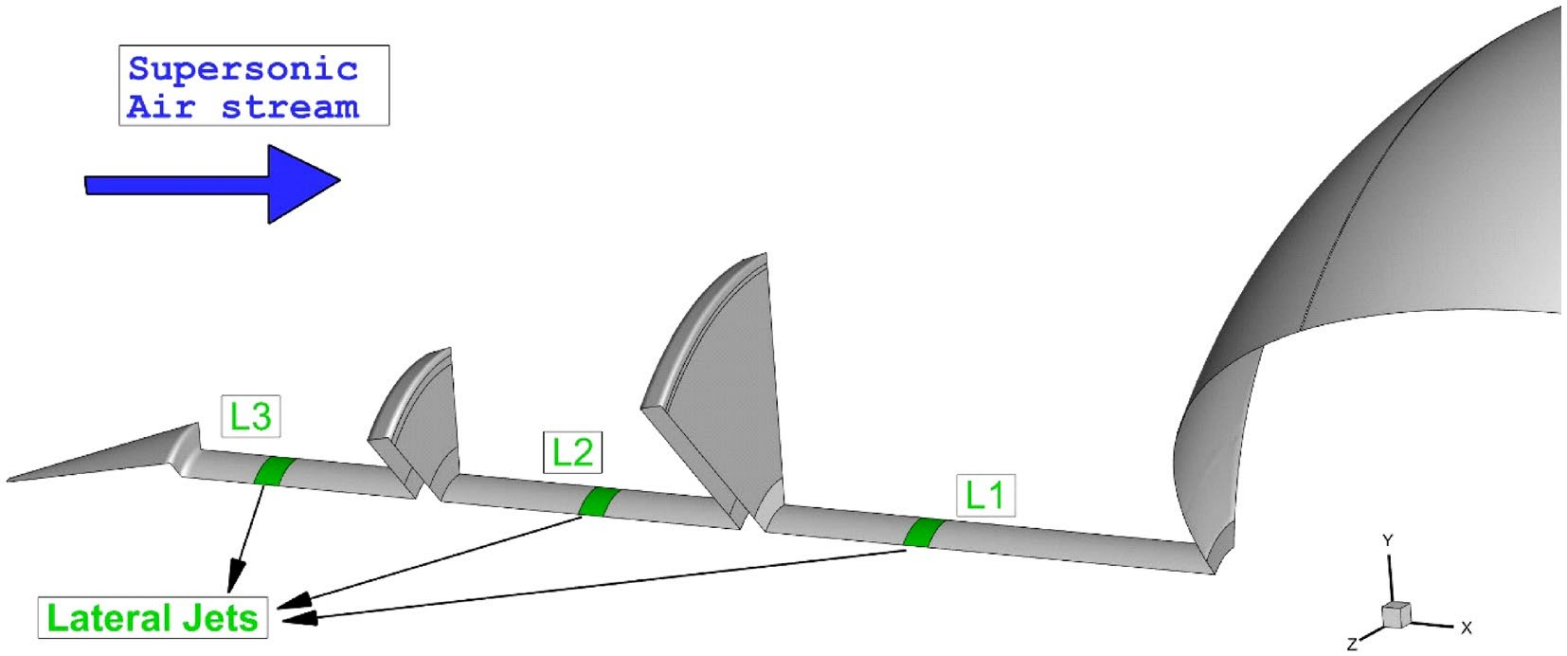
Selected model with proposed injection system.

In this research, the usage of lateral jet for the cooling of the nose cone with multi-row disk (MRD) at high-speed flight is fully investigated (Fig. 1). The influence of jet location and condition on the cooling of the nose cone is investigated by the computational method. The highly compressible flow around the MRD blunt body is simulated and comprehensive flow analysis are presented to find the effective terms for thermal load management of the nose cone. The influence of coolant gas type is investigated by comparing carbon dioxide and helium jet in this investigation.

## Governing equation and numerical method

This study applied RANS equations for modeling of the compressible flow near the nose cone with MRD device^68^. SST turbulence model is applied in the simulation of highly turbulent flow around the nose cone^69^. The flow is assumed ideal gas and species transport equation is also applied since the secondary gases of helium and CO_2_ are used for the cooling in this hybrid technique. Computational fluid dynamic is applied for the simulation of flow around the nose while the coolant gas is released. This technique is popular for simulation of fluid in engineering problems^70,71^. The details of the main governing equations have extensively presented and explained in the previous articles and readers are referred to these resources^72,73^.

**Figure 2:**
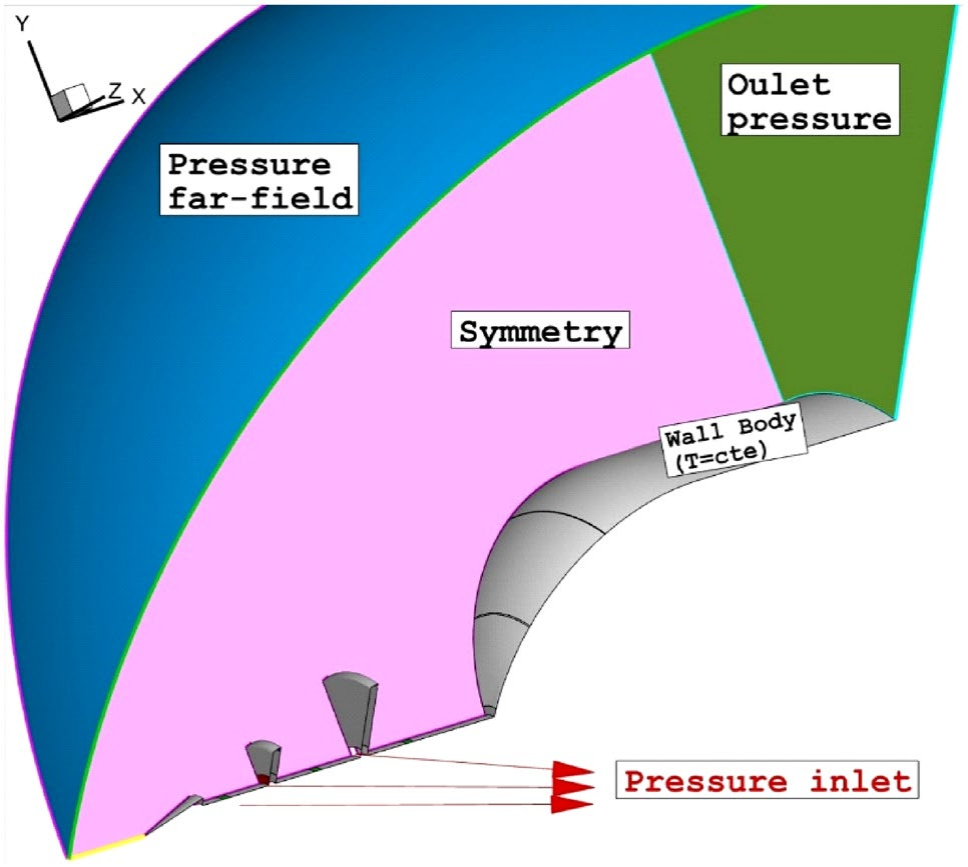
Applied boundary condition.

Applied boundary condition related to the selected model is demonstrated in Fig. 2. Inflow is pressure farfield with M = 5.0, Pinf-2550 and Tinf = 221 K. Helium and carbon dioxide are chosen for as coolant jets with sonic condition at Ts = 300 K. Pressure outlet is extrapolated from the results of inside domain. The spike and main body is assumed wall with constant temperature of 300 K. The length of spike is equal to diameter of the main body^60^.

**Figure 3:**
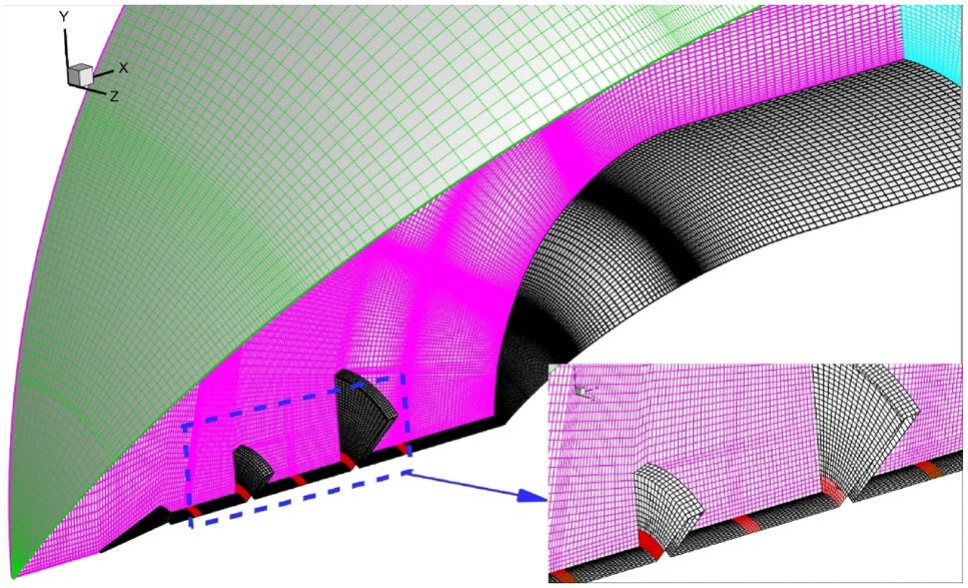
Grid production.

Grid study as the main step for the computational fluid dynamic are done by producing different grids for our models. The number of grid in three directions are change to find optimum model in which results are independent from grid. Figure 3 demonstrated the schematic of produced grid for our model. Structured grid is used since it has more accuracy in the finite volume based approach. Table 1 presented details of grid studies. For grid independency analysis, four grid resolutions are generated and simulated in the first step. Comparison of the heat load on the main body are done for produced grids (Table 1) and it is found that fine grid with 1,628,000 cells.

**Table 1:** Grid details.

Model	Grid number	Average Stanton numb. on blunt cone (θ = 30)	Average Stanton on numb. blunt cone (θ = 60)
Coarse grid	680,000	0.00212	0.00618
Normal grid	960,000	0.00245	0.00637
Fine grid	1,320,000	0.00251	0.00651
Very fine grid	1,680,000	0.00253	0.00653

## Results and discussion

The comparison of experimental and numerical data with our results is done to perform validation. This step is important since it approves the correctness of applied method for the simulation of the chosen case. As presented in ref.^74^, the variation of normalized pressure along the nose agrees reasonably with other methods. The deviation of the archived results from other techniques is not more than 8% in the simple nose cone at supersonic flow.

**Figure 4:**
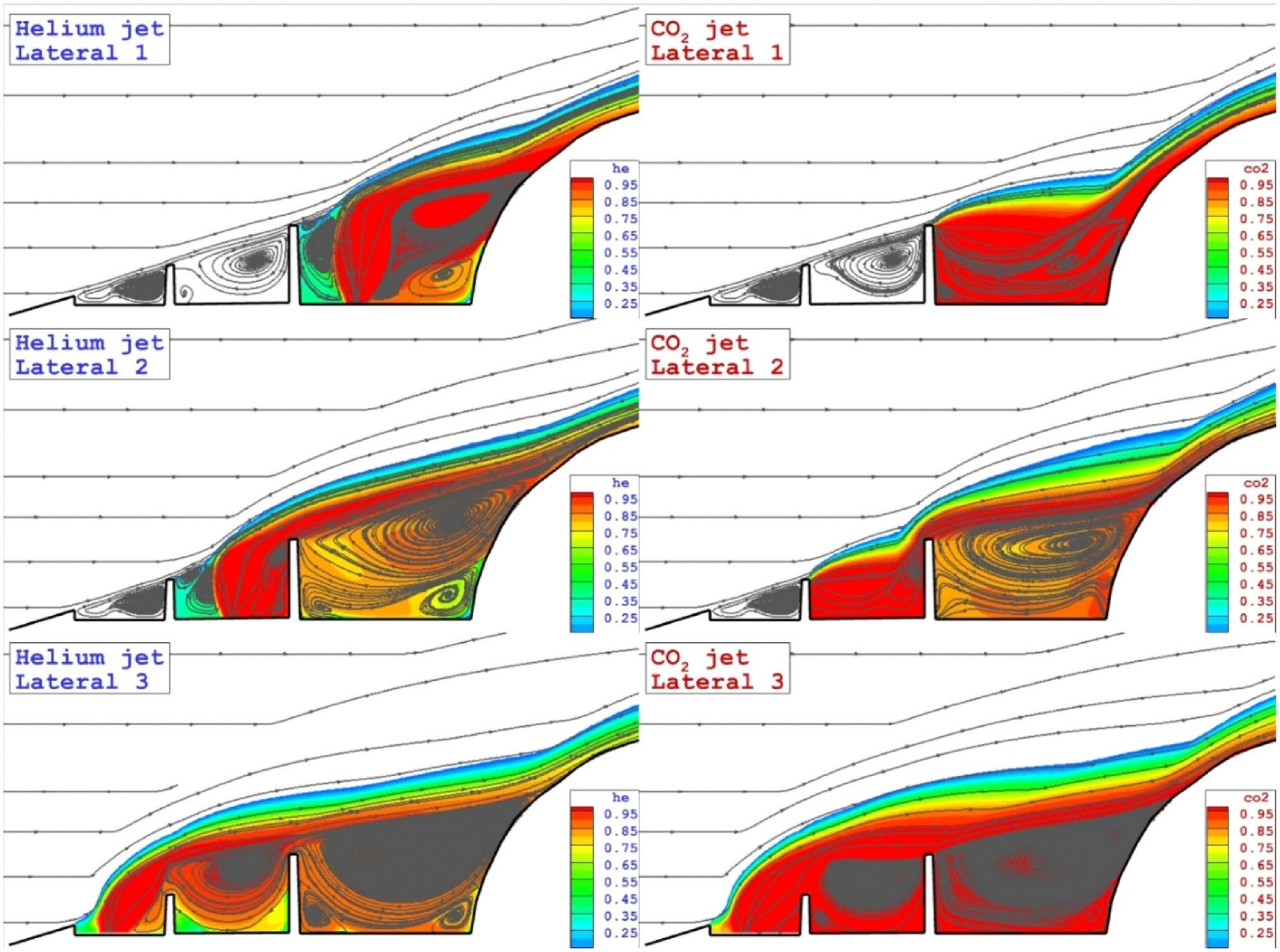
Flow stream and concentration of the different lateral coolant injection systems.

Streamline and coolant distribution for three lateral jets located on the stem of the spike are displayed in Fig. 4. The deflection of the main stream and the diffusion mechanism of Helium and CO_2_ jet in these configurations are noticed in these models. The main effects of these jet locations are on the deflection of main stream while the circulation regime in these model is almost identical. Due to high penetration rate of helium, this gas deflects the bow shock with higher angles.

**Figure 5:**
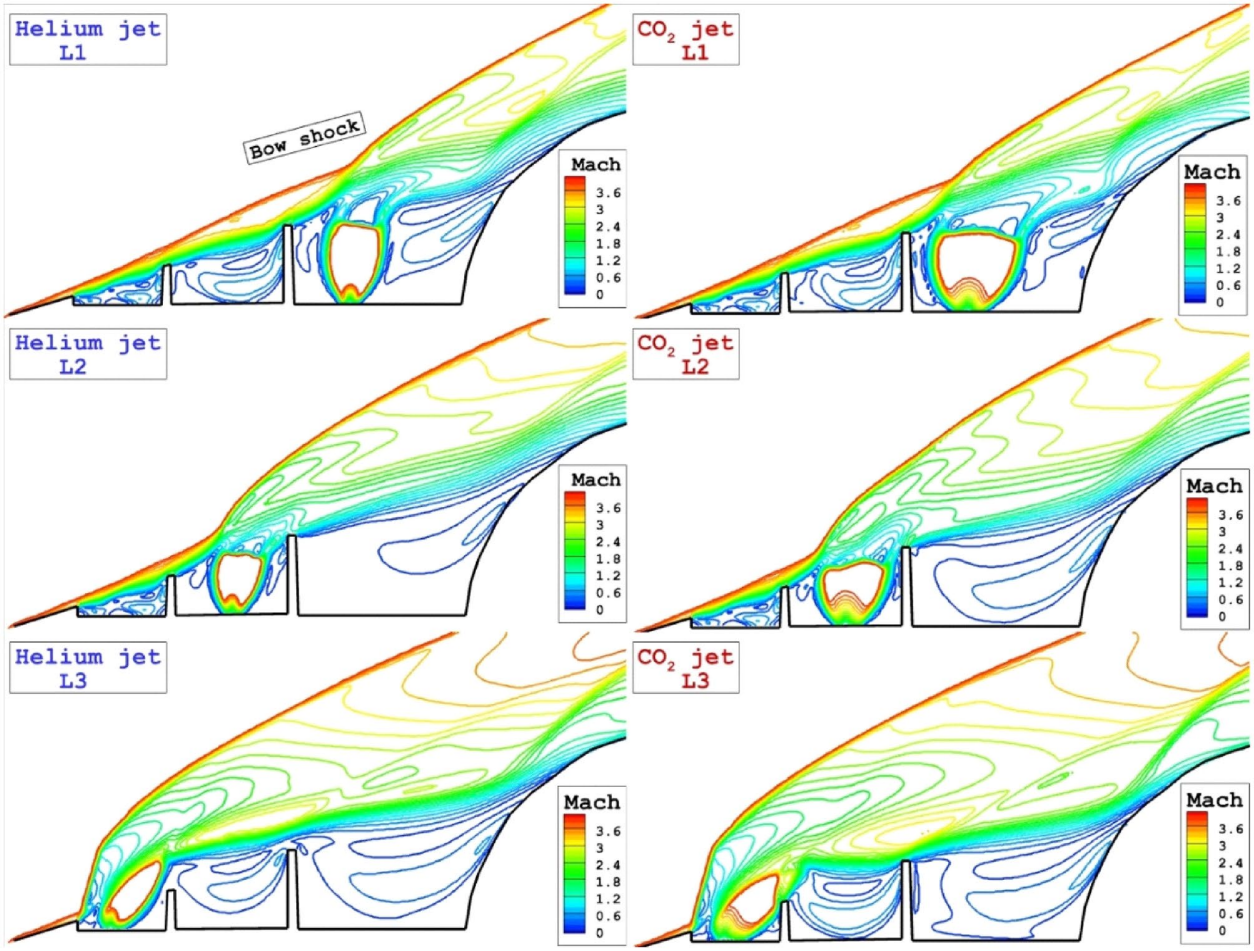
Influence of the different lateral coolant injection systems on shock interactions.

The feature of the shock interactions for lateral injection system are demonstrated in Fig. 5. The main difference on the jet location on the spike is related to the interaction of the separation shock with barrel shock of coolant jet. In fact, this interaction results in deflection of the bow shock and limited the interaction of the separation shock to the main body. As jet location move to the tip of the spike, the angle of the bow shock becomes more and separation layer did not touch the main body. Therefore, the heat transfer decreases on the main body. The main difference of these coolant jet is associated with the shape and size of barrel shock and their effects on the bow shock is almost identical.

**Figure 6:**
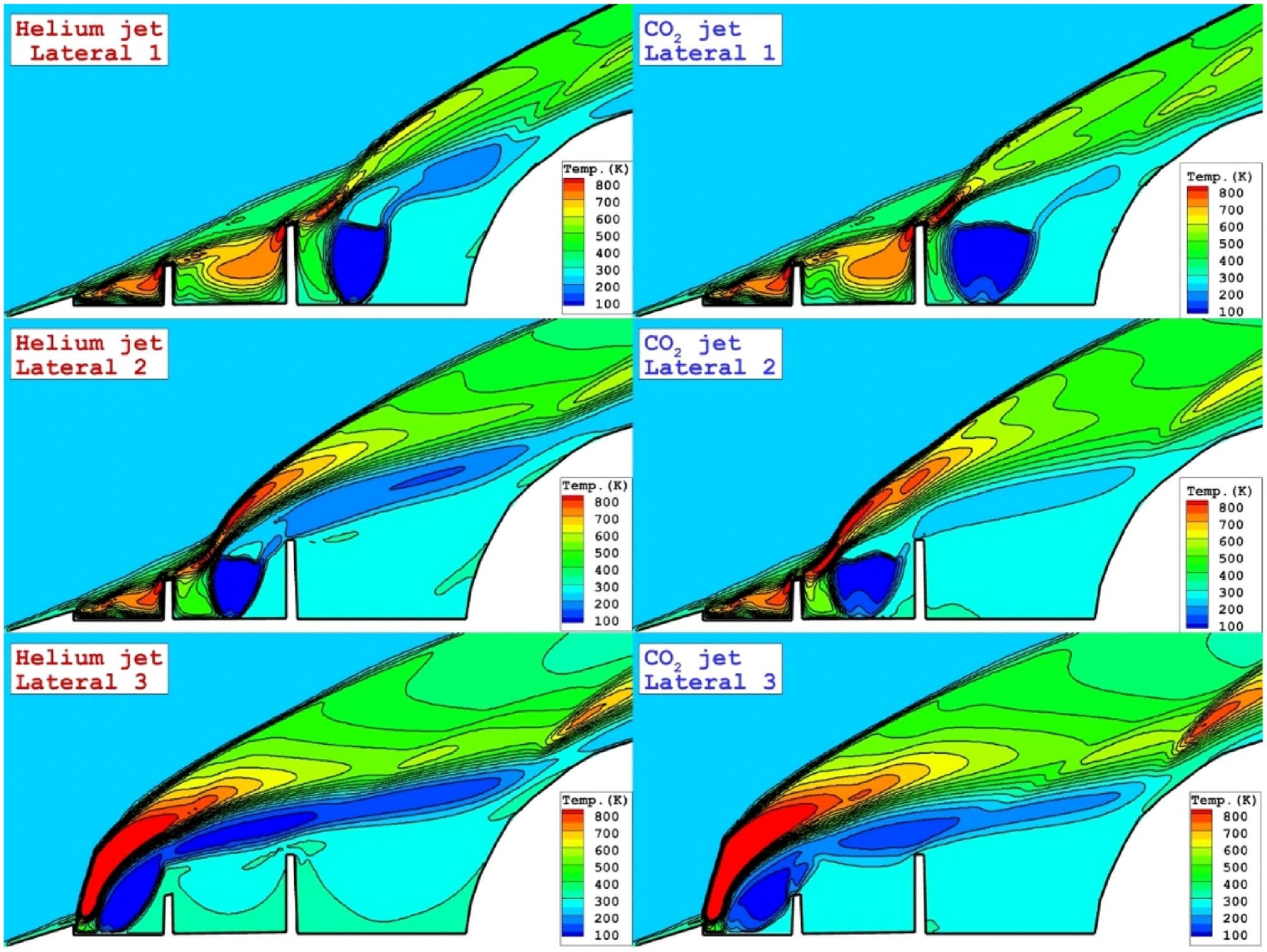
temperature distributions nearby the main body for the different lateral coolant injection systems.

To evaluate the strength of bow and barrel shocks, Fig. 6 demonstrates the temperature contour on the mid-plane for different lateral injection systems. When the lateral injection occurs in the vicinity of the main body, the hot region is nearby the tip of disks where the interaction of the bow shock with disk results in the high entropy region. As coolant injection move to the tip of the spike, the temperature region become restricted between barrel shock and the bow shock and this confirms the high power of bow shock. It is also found that the strength of deflection shock for helium jet is less than that of CO2 jet. Besides, as the coolant jet moves to the main body, more portion of the body is under impacts of the cool fluid.

**Figure 7:**
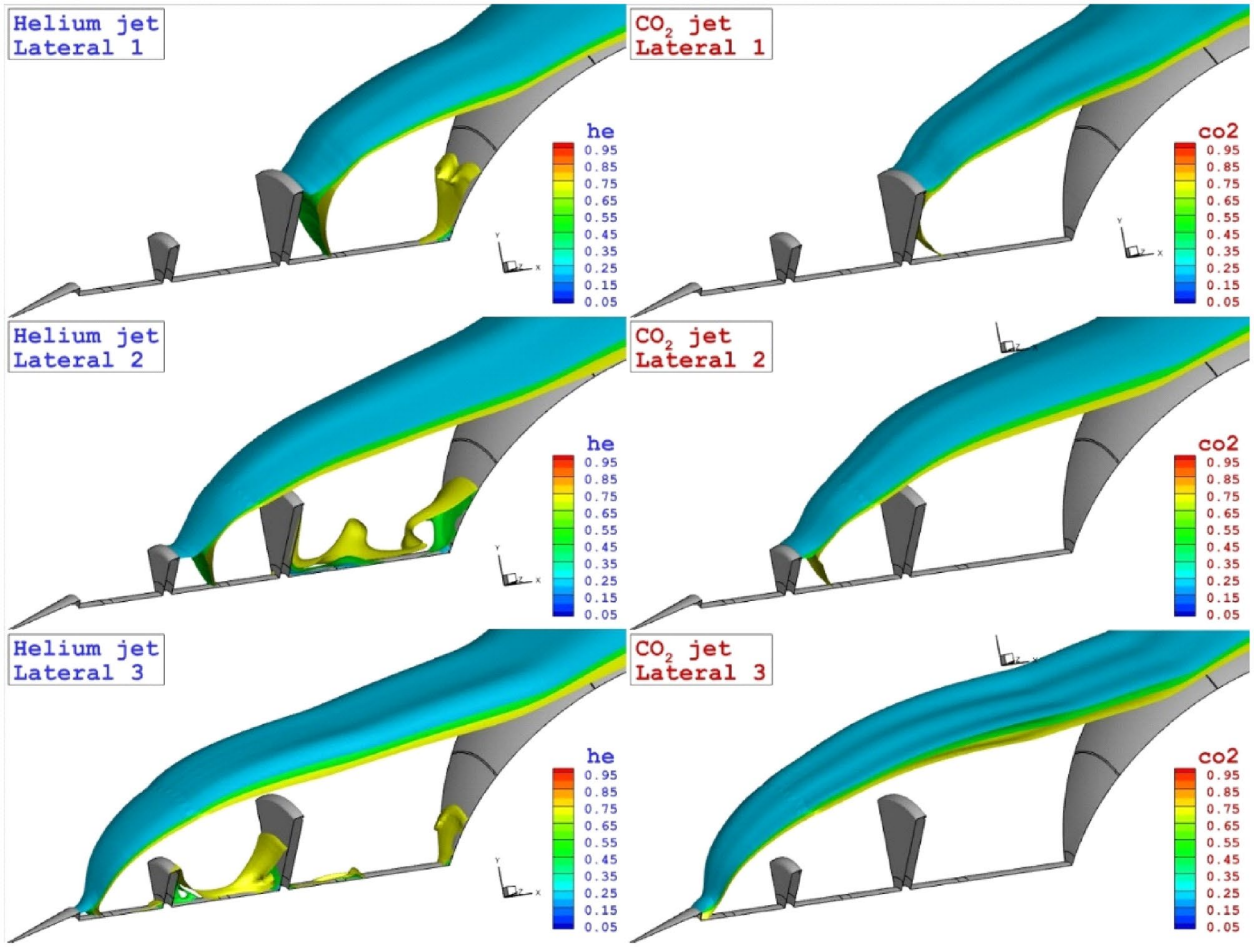
3-D feature of the different lateral coolant injection systems.

**Figure 8:**
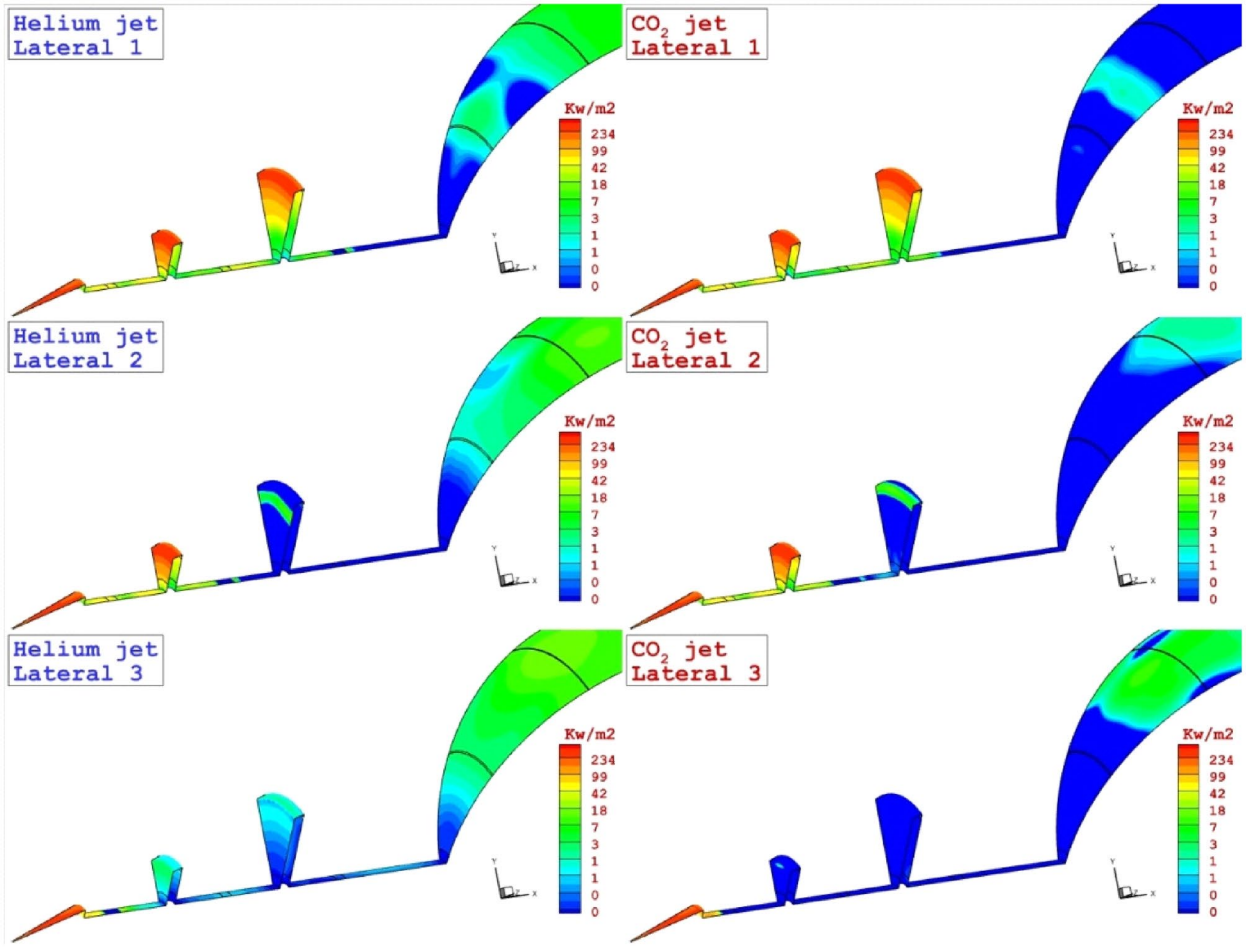
Heat transfer rate on the main body and disk of the different lateral coolant injection systems.

Figure 7 illustrates the three-dimensional feature of the coolant layer to disclose the diffusion of these two gases in different lateral injection systems. Based on the achieved contour, the diffusion of the helium into the main bow shock cases the fluctuation and a segment of coolant diverted into the main body of the nose cone. This effect is noticed on the heat transfer rate displayed in Fig. 8. The heat transfer rate on the disk and main body indicates the diffusion mechanism of the coolant and its effects on the heat load of the nose and disk. As expected, high heat transfer rate occurs on the tip of the disk and this is because of shock deflection. Effects of coolant location is also noticed on the heat transfer of the main body.

**Figure 9:**
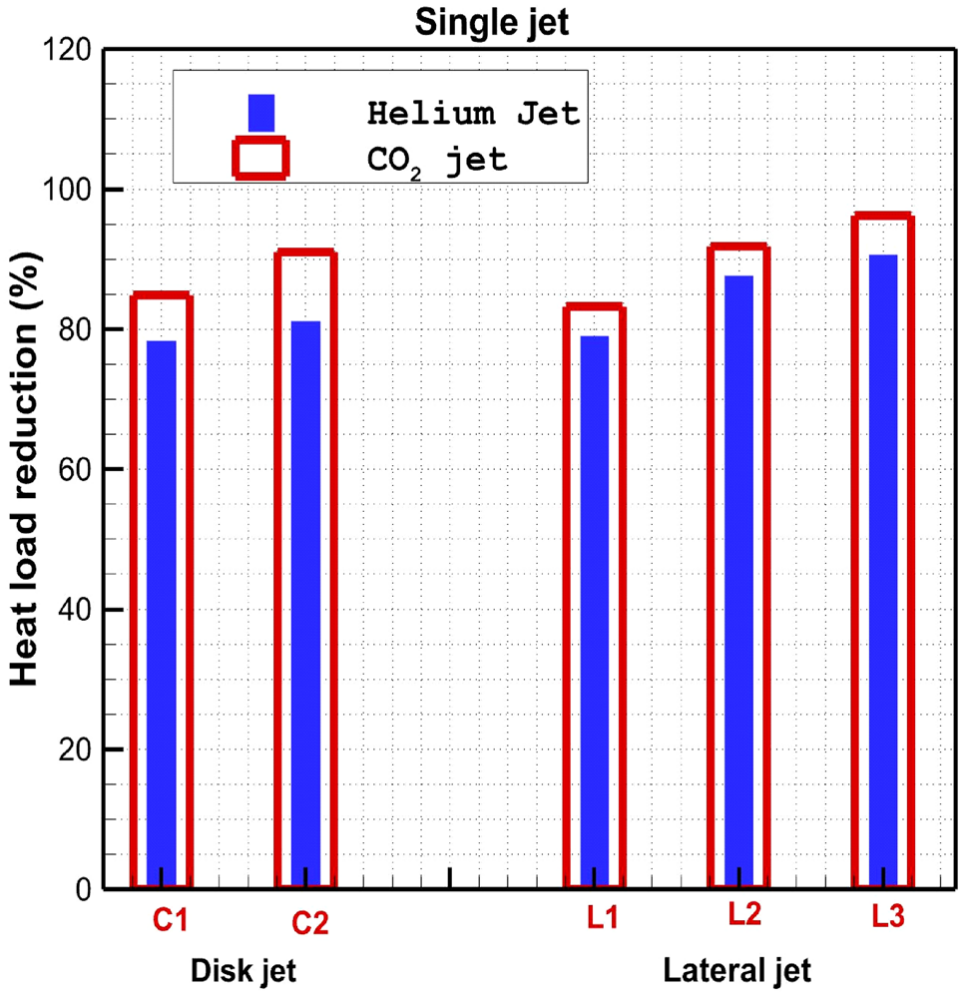
Comparison of heat load reduction of the different lateral coolant injection systems.

Figure 9 demonstrates the effect of the different lateral coolant injection systems on the total heat load reduction on the main body and spike assembly. Obtained data indicates that the injection of CO_2_ jet is more efficient than Helium for cooling of the body and spike assembly. In fact, this is due to the shield effects of the CO_2_ gas since it has lower diffusivity than helium.

## Conclusion

This study tries to investigate the importance of the lateral jet for the thermal management of the nose cone with MRD flying at hypersonic flow. Three-dimensional model is used for the investigation of the flow and heat transfer near the nose cone and spike assembly. Flow analysis and coolant gas distribution are compared for two coolant gas types of helium and carbon dioxide. The influence of the coolant gas on the compression shock and bow shock near spike and main body. Mechanism of cooling in different jet locations is also investigated to achieve the optimum configuration for the thermal load reduction of the nose cone. Our results show that deflation of the main blow shock by the coolant jet near the spike tip has great impacts on the reduction of aerodynamic heating.

## Data Availability

The datasets used and/or analysed during the current study available from the corresponding author on reasonable request.
